# Nutrient amendments enrich microbial hydrocarbon degradation metagenomic potential in freshwater coastal wetland microcosm experiments

**DOI:** 10.1128/aem.01972-24

**Published:** 2024-12-09

**Authors:** Katie E. Howland, Jack J. Mouradian, Donald R. Uzarski, Michael W. Henson, Donald G. Uzarski, Deric R. Learman

**Affiliations:** 1 Institute for Great Lakes Research, CMU Biological Station, and Department of Biology, Central Michigan University 172740 , Mount Pleasant, Michigan, USA; 2 Department of Biological Sciences, Northern Illinois University 166942 , DeKalb, Illinois, USA; Washington University in St. Louis, St. Louis, Missouri, USA

**Keywords:** microbial biodegradation, metagenomic, monoaromatic hydrocarbons, polycyclic aromatic hydrocarbons, BTEX

## Abstract

Biostimulating native microbes with fertilizers has proven to be a highly effective strategy to speed up biodegradation rates in microbial communities. This study investigates the genetic potential of microbes to degrade light synthetic crude oil in a freshwater coastal wetland. Experimental sediment microcosms were exposed to a variety of conditions (biological control, a light synthetic crude oil amendment, and light synthetic crude oil with nutrient amendment) and incubated for 30 days before volatile organic compounds (BTEX) were quantified and DNA was sequenced for metagenomic analysis. The resulting DNA sequences were binned into metagenome-assembled genomes (MAGs). Analyses of MAGs uncovered a 13-fold significant increase in the abundance of rate-limiting hydrocarbon degrading monooxygenases and dioxygenases, identified only in MAGs from the light synthetic crude oil with nutrient amendments. Further, complete degradation pathways for BTEX compounds were found only in MAGs resulting from the light synthetic crude with nutrient amendment. Moreover, volatile organic compounds (BTEX, cyclohexane, and naphthalene) analyses of microcosm sediments in the presence of nutrients documented that benzene was degraded below detection limits, toluene (98%) and ethylbenzene (67%) were predominantly reduced within 30 days. Results indicate that the genetic potential to degrade BTEX compounds in this freshwater wetland can be linked to the functional potential for bioremediation. BTEX compounds are typically more recalcitrant and tougher to degrade than alkanes. This study demonstrated that stimulating a microbial community with nutrients to enhance its ability to biodegrade hydrocarbons, even in a relatively nutrient-rich habitat like a freshwater wetland, is an effective remediation tactic.

**IMPORTANCE:**

The impact of oil spills in a freshwater aquatic environment can pose dire social, economic, and ecological effects on the region. An oil spill in the Laurentian Great Lakes region has the potential to affect the drinking water of more than 30 million people. The light synthetic crude oil used in this experimental microcosm study is transported through an underground pipeline crossing the waterway between two Laurentian Great Lakes. This study collected metagenomic data (experiments in triplicate) and assessed the quantity of BTEX compounds, which connected microbial degradation function to gene potential. The resulting data documented the bioremediation capabilities of native microbes in a freshwater coastal wetland. This study also provided evidence for this region that bioremediation can be a viable remediation strategy instead of invasive physical methods.

## INTRODUCTION

Microbial communities are vital players in hydrocarbon degradation. Past crude oil spills, including Deepwater Horizon and Exxon Valdez, have noted that microbial activity significantly reduces environmental impact relative to physical cleanup methods (1). To date, there is a plethora of knowledge related to hydrocarbon degradation in marine environments (1–6), yet limited knowledge is available on how freshwater microbial communities will respond to hydrocarbon exposure (7–10). Many freshwater studies have also been limited to single gene analysis, focusing on community ecology (e.g., 16S rRNA) (7–10). Thus, there is a need for additional studies in freshwater habitats as, according to the U.S. Environmental Protection Agency (EPA), oil spills happen more often in freshwater environments than marine environments and are usually more detrimental to the environment (11).

Microorganisms possessing the ability to degrade hydrocarbons are said to be ubiquitous in the environment (12). Previous studies have determined that in the presence of hydrocarbons, microbial communities will shift, resulting in a greater number of hydrocarbon degradation genes, as well as known hydrocarbon-degrading taxa, most notably *Gammaproteobacteria* and *Alphaproteobacteria* (2, 6, 10). *Pseudomonadales*, *Burkholderiales*, and *Xanthomonadales*, all orders within Gammaproteobacteria, have been characterized by their ability to degrade various hydrocarbons (13–15). While bacteria have the potential to degrade hydrocarbons to gain energy for growth (12, 15–17), very few organisms possess the enzymes necessary to degrade crude oil completely (18). Complete biodegradation of hydrocarbon fractions within crude oil requires a diverse population of bacteria possessing a wide range of enzymatic capabilities (18–20). Many studies have documented that the complete degradation of hydrocarbons often involves a consortium of bacteria working together (3, 18, 20–22), with one study (14) concluding that <0.5% of genomes studied with hydrocarbon degradation contain all the genes and enzymes necessary to degrade hydrocarbons completely.

Crude oil is diverse, containing a mixture of alkanes, monocyclic aromatic hydrocarbons (MAHs), and polycyclic aromatic hydrocarbons (PAHs) (23), with MAHs and PAHs being the most challenging fractions to degrade (24, 25). Additionally, MAHs like benzene, toluene, ethylbenzene, and xylene (BTEX) and PAHs like naphthalene are considered highly toxic and relatively more recalcitrant (24). Microbial degradation of MAHs and PAHs, like BTEX and naphthalene, are activated by monooxygenases and dioxygenases. Like all biological pathways, hydrocarbon degradation requires essential genes to activate pathways (14, 26). Thus, monooxygenases and dioxygenases are rate-limiting in the degradation of recalcitrant MAHs (BTEX) and PAHs (naphthalene) (14, 26). Alkanes are generally easier to degrade than their more recalcitrant aromatic counterparts (24, 27), with several studies reporting saturated alkanes as the preferred hydrocarbon for biodegradation (28, 29). MAHs and PAHs also pose both an ecological and human health risk. Benzene and ethylbenzene are carcinogenic, while toluene and xylene pose other adverse effects (30). Moreover, the solubility of the BTEX components of the light synthetic crude oil allows for expeditious contamination of soil (31), thus emphasizing the need for metagenomic studies such as the current study.

Monooxygenases and dioxygenases catalyze aerobic hydrocarbon degradation of BTEX and naphthalene (19, 23). Initial degradation of benzene, toluene, and xylene is often initiated by monooxygenases, including *tmo*, *tomA*, and *dmp* genes (32, 33). Ethylbenzene degradation is usually catalyzed by the dihydroxylation involving naphthalene, ethylbenzene, and biphenyl dioxygenases (13, 34, 35). Naphthalene degradation is activated by naphthalene dioxygenases (NDOs) (3, 36, 37). NDOs have diverse potentials and degrade various substrates, including ethylbenzene (13), naphthalene (38), and xylene (39).

This study focused on the degradation of recalcitrant MAHs and PAHs, like BTEX and naphthalene, which are components in light synthetic crude oil used in this study (10). An existing underwater light synthetic crude oil pipeline crosses the hydrological connection (Straits of Mackinac) between two of the Laurentian Great Lakes, Lakes Huron, and Michigan (USA) (40, 41). This oil pipeline transports approximately 23 million gallons of light synthetic crude oil daily through this area, and due to the pipeline’s age, there is a concern about a potential spill in this unique ecosystem (40). Consequently, the freshwater wetland studied here lies in the proposed path (40) if a spill occurs. The Laurentian Great Lakes contain approximately 80% of surface freshwater in North America and nearly 18% of the world’s surface freshwater (42, 43). The Laurentian Great Lakes also provide drinking water to over 30 million people and are also of great economic importance, as they account for approximately $5 trillion annually and support over 50 million jobs (42, 43). Wetlands are vital as they act as a filter or a barrier, preventing pollutants and toxicants from entering the surrounding watershed. They are also hubs of biodiversity, providing a habitat to numerous endangered species and plants (44, 45). Previous studies have identified crude oil contamination persisting in freshwater wetlands for more than five years (46, 47), highlighting the need for metagenomic studies focusing on freshwater wetland sediment communities.

To examine the genetic potential to degrade hydrocarbons in a freshwater coastal wetland, a 30-day sediment microcosm experiment was conducted with biological control, a light synthetic crude oil amendment, and light synthetic crude oil with a nutrient amendment (which includes replicates). This study builds upon previous research that determined the presence of light synthetic crude oil caused a shift in community (beta diversity) and an increase in taxa of known hydrocarbon degraders in freshwater habitats (9, 10). Further, previous studies have shown that biostimulation, specifically the addition of nutrients, speeds up the process of biodegradation of hydrocarbons (1, 19, 48). Shotgun metagenomics and bioinformatics were employed to delve into the genes, taxa, and pathways involved in hydrocarbon degradation to examine gene potential in the experimental microcosms. To further identify rate-limiting activation genes, a curated set of HMMs that specialize in hydrocarbon (MAH, PAH, and alkanes) degradation was used to annotate MAGs (49). Moreover, volatile organic compound (VOC) analysis of the resulting microcosm and statistical analyses will further elucidate the potential of native microbial communities to degrade light synthetic crude oil. Taken together, data from the present study provided insight into the genetic potential of hydrocarbon degradation by the microbial community.

## MATERIALS AND METHODS

### Sediment sampling and microcosm setup

Sediment and water samples were collected on 10 July 2020, from a coastal wetland near St. Ignace, MI, USA (45.84777N, −84.73888W). The sediments were collected from multiple cores within the wetland using a previously established protocol (10). In brief, several centimeters of sediments were obtained via a push corer, with multiple cores collected from the site. The plunger was slowly pushed out until only 3 cm remained (representing surface sediments) to remove the sediments from the corer. The top 3 cm were placed in a Whirl-Pak bag and transported to Central Michigan University (CMU) in a cooler with ice. Water was collected in a 1 L bottle next to each core (approximately 10 ft from where sampling took place) before core sampling to reduce the number of suspended solids in the sample and was transported similarly to the sediments. Upon arrival at CMU, sediments and water were immediately stored at 4°C overnight.

Experimental microcosms were set up the day after the samples were collected (11 July 2020), following a previously described protocol with some modifications (10). In brief, triplicate experiments of three amendments were constructed: a biological control (without amendments), a light synthetic crude oil amendment, and light synthetic crude oil with a nutrient amendment. Replicate 1 for each treatment received sediments and water from core 1, replicate 2 from core 2, and replicate 3 from core 3. Each consisted of two-time points (days 0 and 30) that were destructively sampled. The biological controls comprised approximately 50 g of sediments covered with 45.625 mL of lake water. The crude amendment was set up with approximately 50 g of sediments, 43.5 mL of lake water, and 2.125 mL of light synthetic crude oil donated by Enbridge (Calgary, AB). The third treatment consisted of 50 g of sediments, 35 mL of lake water, 2.125 mL of light synthetic crude oil donated by Enbridge (Calgary, AB), and 8.5 mL of 10× Bushnell-Haas media (50) (Table S1). The final weight of each jar was approximately 95.5 g.

On 11 July 2020, microcosm jars labeled day 0 were stored at −20°C, and jars labeled day 30 were placed in an environmental growth chamber to simulate *in situ* conditions. The mesocosm was set at 22°C, and light intensity was set at 700 footcandles with lights turning on at 06:00 and off at 21:30 to mimic sunrise and sunset (10). Microcosms were removed from the environmental growth chamber on 10 August 2020, and stored at – 20°C for further analysis.

### DNA extraction, sequencing, and analysis

DNA was extracted using DNeasy Powersoil Kit from Qiagen (Valencia, CA, USA) following a previously established protocol with a few refinements (51). Briefly, three extractions for each of the three replicates of each treatment (crude and crude fertilizer) and control was taken. Each replicate’s extractions were then pooled and concentrated using Zymo clean and concentrate kit (Irvine, CA, USA). DNA was quantified using a Qubit 2.0. DNA was sequenced on Illumina Hi-Seq 4000 (PE 150 bp) at Michigan State University Research Technology Support Facility (Lansing, MI, USA), using Illumina TruSeq Nano DNA library preparation, producing 720,680,807 reads. Raw DNA sequencing reads are publicly available at NCBI GenBank under BioProject number PRJNA761709. Read quality was checked before downstream analysis using FastQC (52). Paired-end reads were evaluated with Trimmomatic v0.39 with default settings to trim adapters and primers (53). Forward and reverse reads were interleaved using khmer v2.1.1 (54).

### Metagenomic assembly, binning, and annotation

A previously established protocol was used for assembly, binning, and annotation with slight alterations (55). Triplicate replicates for each treatment or control were co-assembled at Michigan State University’s high-performance computing center using MEGAHIT v1.12 (56). Quast v5.0.0 was used to check the quality of read-based co-assemblies (57). MEGAHIT co-assemblies were submitted to IMG for annotation (58) (Table S2).

To assemble metagenome-assembled genomes (MAGs), co-assemblies were first mapped with BWA 0.7.17 (59), converted to bam files using SAMtools 1.11 (60), and binned using Metabat2 v2.15 (61) with no added flags. The resulting MAGs were quality-checked for completeness and contamination using CheckM v1.1.3 (62). After manually refining bins using Anvi’o (v7) (63), MAGs were rechecked for quality using CheckM v1.1.3. MAGs ≥75% complete with ≤3.5% contamination were used for all downstream analyses. Taxonomic classification of MAGs was obtained using GTDB-tk v1.3.0 classifier (64). MAGs were annotated first with MagicLamp (65), with an e-value threshold of 1E−10, using the CANT-HYD hydrocarbon degradation database to search for novel and understudied hydrocarbon degradation activation genes (49). All HMMs provided by the CANT-HYD database hit to a single gene, except MAH⍺,β which represents a broad set of genes, MAH⍺ (genes *tcbA*, *lpbA*, *bnzA*, and *bphA*) and MAHβ (genes *tcbAb*, *todC2*, and *bphAb*) capable of degrading several substrates. Additionally, *almA* was found to be highly diverse by a previous study (66). Therefore, it was split into two HMMs, groups I and III (49), to represent the classification system. Curated MAGs were annotated using Anvi’o (v7) with KEGG orthologs modules, and then putative metabolic pathways were reconstructed using the command “anvi-estimate-metabolism” (63, 67–71).

To assess the abundance of organisms and quantify gene annotations, reads were competitively recruited to MAGs using a previously described protocol with slight alterations (72). First, reads were filtered for quality with Illumina utils v2.6 (73). MAGs for each amendment and control were concatenated to allow for a competitive recruitment of reads for MAG against MAG. Raw reads were mapped to prospective MAGs using bowtie2 v2.4.5 (74), and resulting sam files were converted to bam files with SAMtools v1.14 (60). Reads were filtered using CoverM v0.7.0 to remove low-quality mappings using a minimum identity of 95% and minimum read alignment of 75% (75). A count table for each amendment and control was generated using get_count_table.py script (76). The number of reads that hit each MAG was then transformed to reads per kilobase million (RPKM) to normalize MAG data and obtain a normalized abundance of MAGs (Table S3). MAGs resulting from light synthetic crude oil and light synthetic crude oil with nutrient amendments were competitively mapped back to control to observe changes in taxonomic composition between treatments and control.

### Chemical analysis

Triplicate samples of sediments were analyzed at TRACE Analytical Laboratories, INC (Muskegon, MI, USA) for VOCs and sediment pH. Briefly, 30–40 g of sediments was sent to TRACE analytical (Muskegon, MI, USA), where the EPA-8260D (77) method was employed to measure concentrations of benzene, toluene, ethylbenzene, total xylene, cyclohexane, and naphthalene using a dry weight. Dry weight was obtained using method ASTM D2974-07a (78). Method EPA-9045D (79) was utilized to determine pH.

### Statistical analyses

Statistical analyses were performed with R v4.1.1, with packages tidyverse, coin, and rstatix (80). A Friedman analysis was performed in package tidyverse on sediments from microcosms to determine if there was a significant difference in the concentration of VOCs between days and day 30 microcosms, with a significant threshold of *α* = 0.05 (81). The Friedman test was set up as a global test where each block consisted of six replicates (three replicates for each time point) and was grouped by component (benzene, toluene, ethylbenzene, xylene, and cyclohexane). To run the Friedman test, all true replicates needed values; therefore, the analysis was not conducted on naphthalene. A paired Wilcoxon signed-rank test was performed with packages coin and rstatix to compare the abundance of hydrocarbon degradation activation genes in each amendment relative to the control, with a significant threshold of *α* = 0.05.

## RESULTS

### Metagenomes and MAGs statistics

DNA sequencing resulted in a total of over 7 million 150 bp paired-end reads. The resulting DNA sequencing reads from triplicate samples for each microcosm treatment were co-assembled, resulting in over 240,000,000 contigs with a length above 10,000 bp (Table S2). Co-assemblies resulted in *N*_50_ values of 935, 953, and 992 for control, crude amendment, and light synthetic crude with nutrient amendment, respectively. Binning of metagenomic data resulted in 122 MAGs meeting high and medium standards (82). Of these MAGs, 37 were selected for downstream analyses as they had a completion score of ≥75% with ≤3.5% contamination.

### Abundance of hydrocarbon degradation gene potential within MAGs

**Fig 1:**
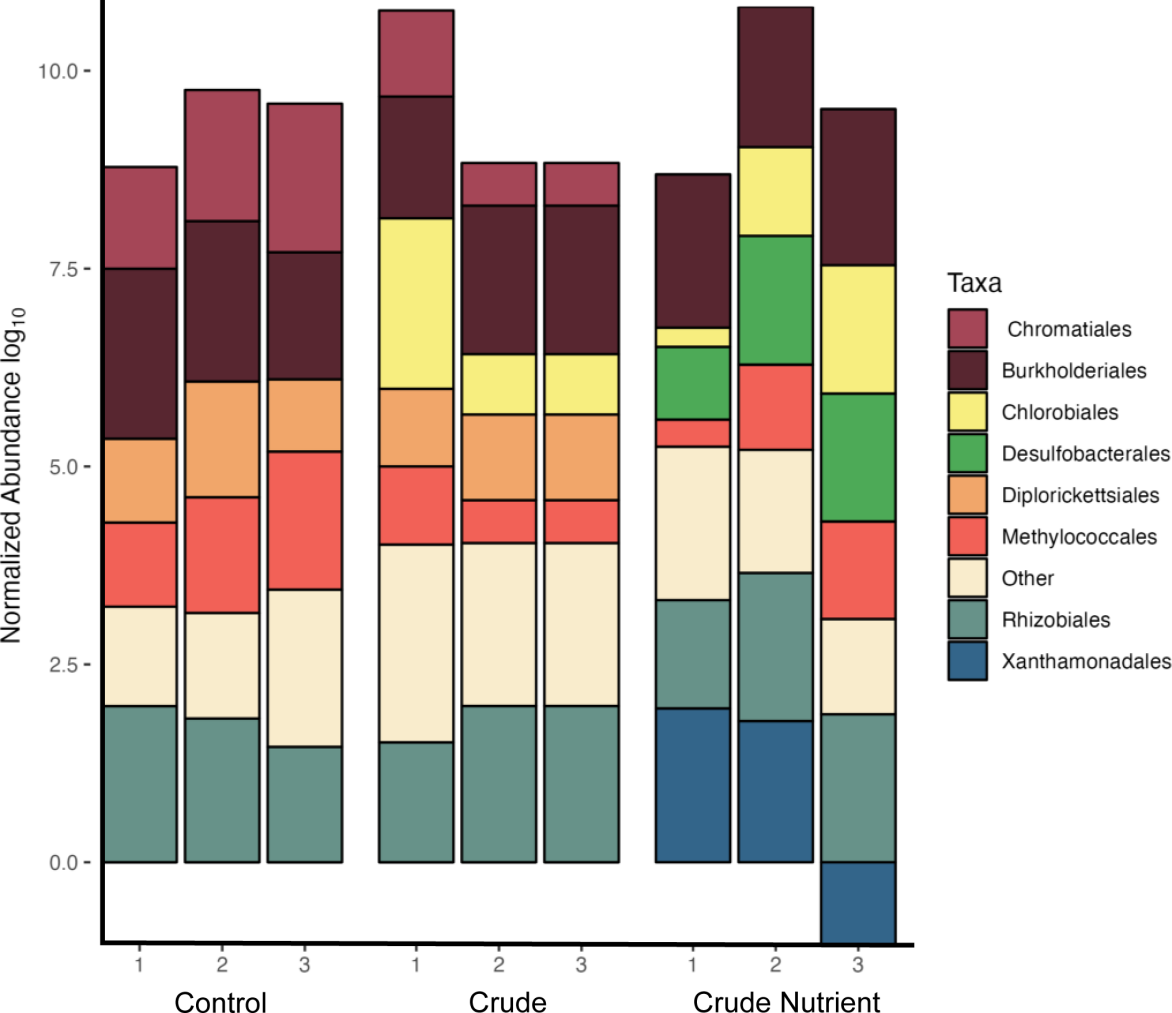
Taxonomic composition and log_10_ normalized abundance of MAGs ≥75% complete with ≤3.5% contamination. Normalized abundance was calculated by RPKM values of selected MAGs for each replicate of amendment and control (RPKM = number of reads/(genome length/1,000 * total number of reads/1,000,000).

Of the 37 high-quality MAGs, 19 contained hydrocarbon degradation genes involved in the aerobic degradation of aromatic compounds. Relative to the MAGs from the control treatment, MAGs from the light synthetic crude oil with nutrient amendment had an increase in hydrocarbon degradation pathway activation genes (Table 1). The light synthetic crude oil with nutrient amendment had a 9-fold increase in alkane (*P* = 0.049) and a 13-fold (*P* = 0.003) increase in aromatic hydrocarbon degradation genes relative to the control treatment. In the experimental condition with only light synthetic crude oil amendment, there is a ninefold increase (*P* = 0.005) in the abundance of aromatic hydrocarbon activation genes relative to the control. There was a non-significant difference in the number of alkane and anaerobic hydrocarbon activation genes between control and light synthetic crude oil amendment (*P* = 0.163 and *P* = 0.05). Competitive mapping (RPKM) of sequencing reads from MAGs within the control and experimental treatments documented changes in taxonomic abundances (Fig. 1). The relatively dominant order identified in the control was Burkholderiales and Rhizobiales (Fig. 1). The addition of light synthetic crude shifted the highest taxonomic abundance to mainly Chlorobiales and Rhizobiales (Fig. 1). Amending the experiments with light synthetic crude oil and nutrients documented Burkholderiales as the relatively dominant order, and this condition also identified Xanthomonadales, which was not found in other experimental conditions (Fig. 1).

**TABLE 1:** Total HMM gene predictions for genes involved in initial activation of both aerobic and anaerobic alkane and aromatic hydrocarbon degradation in MAGs ≥75% complete with ≤.5% contamination

	Gene	Control^*a*^	Light synthetic crude oil^*b*^	Light synthetic crude oil with nutrients^*c*^
Aerobic alkane	*pBmoA*	1	0	1
	*pBmoC*	1	0	1
	*pBmoB*	1	0	1
	*alkB*	1	5	10
	*Cyp153*	4	6	8
	*almA^d^*	1	0	1
	*almA^e^*	0	1	1
	*ladAβ*	0	3	3
	*ladA⍺*	0	4	4
	*ladB*	0	10	11
	*prmA*	0	1	2
	*prmC*	0	0	0
	*bmoX*	0	1	2
Aerobic aromatic	(*MAH⍺) tcbA, lpbA, bnzA, bphA*	0	8	15
	(*MAHβ) tcbAb, todC2, bphAb*	0	5	12
	*tmoA_*	0	0	0
	*tmoE*	0	1	2
	*tomA4*	0	0	1
	*tomA1*	0	1	2
	*tomA3*	0	0	0
	*tmoB*	0	1	2
	*dszC*	5	33	35
	*dmpO*	2	8	19
	*non_ndoB*	1	6	7
	*ndoB*	0	5	9
	*ndoC*	0	1	1
Anaerobic alkane	*ahyA*	2	4	5
	*assA*	0	0	0
Anaerobic aromatic	*abcA_2*	0	1	2
	*abcA_1*	5	8	11
	*ebdA*	4	8	9
	*cmdA*	16	24	27
	*bssA*	0	1	1
	*K27540*	0	0	0
	*nmsA*	2	1	1

^*a*^ There was a non-significant difference in the amount of alkane (*P* = 0.163) and anaerobic (*P* = 0.050) hydrocarbon activation genes between control and light synthetic crude oil amendment (Wilcoxon signed-rank test).

^*b*^ There was a significant difference in the abundance of aromatic hydrocarbon activation genes between the control and light synthetic crude oil amendment (*P* = 0.005) (Wilcoxon signed-rank test).

^*c*^ There was a large significant difference between control and light synthetic crude oil with nutrient amendment in alkane (*P* = 0.049), aromatic (*P* = 0.004), and anaerobic (*P* = 0.042) gene potential (Wilcoxon signed-rank test).

^*d*^ Group III of the *almA* gene.

^*e*^ Group I of the *almA* gene.

**Fig 2:**
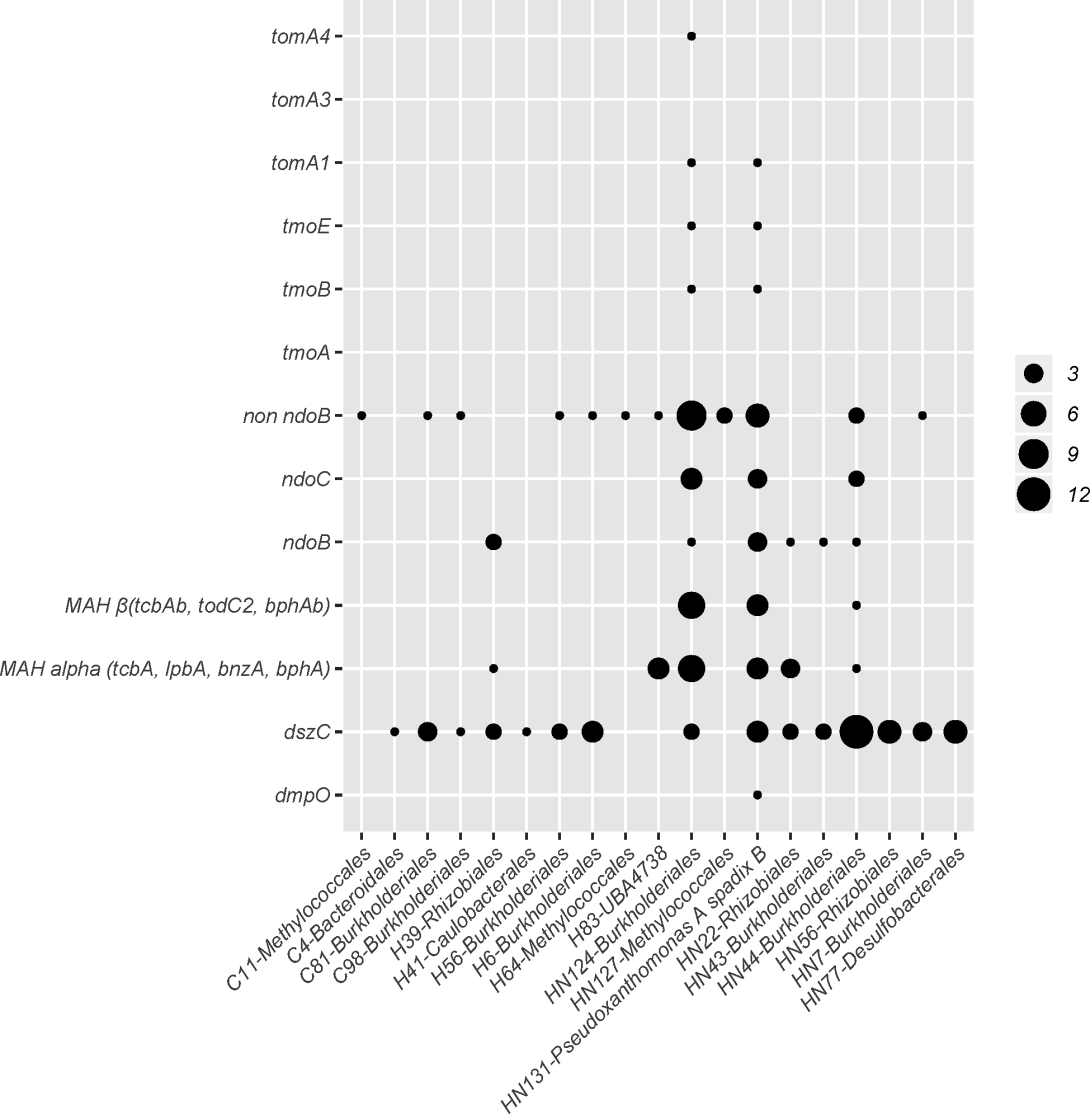
Annotation of MAGs ≥75% complete with ≤3.5% contamination where each dot represents the number of HMM hits displaying hydrocarbon activation gene degradation potential.

**Fig 3:**
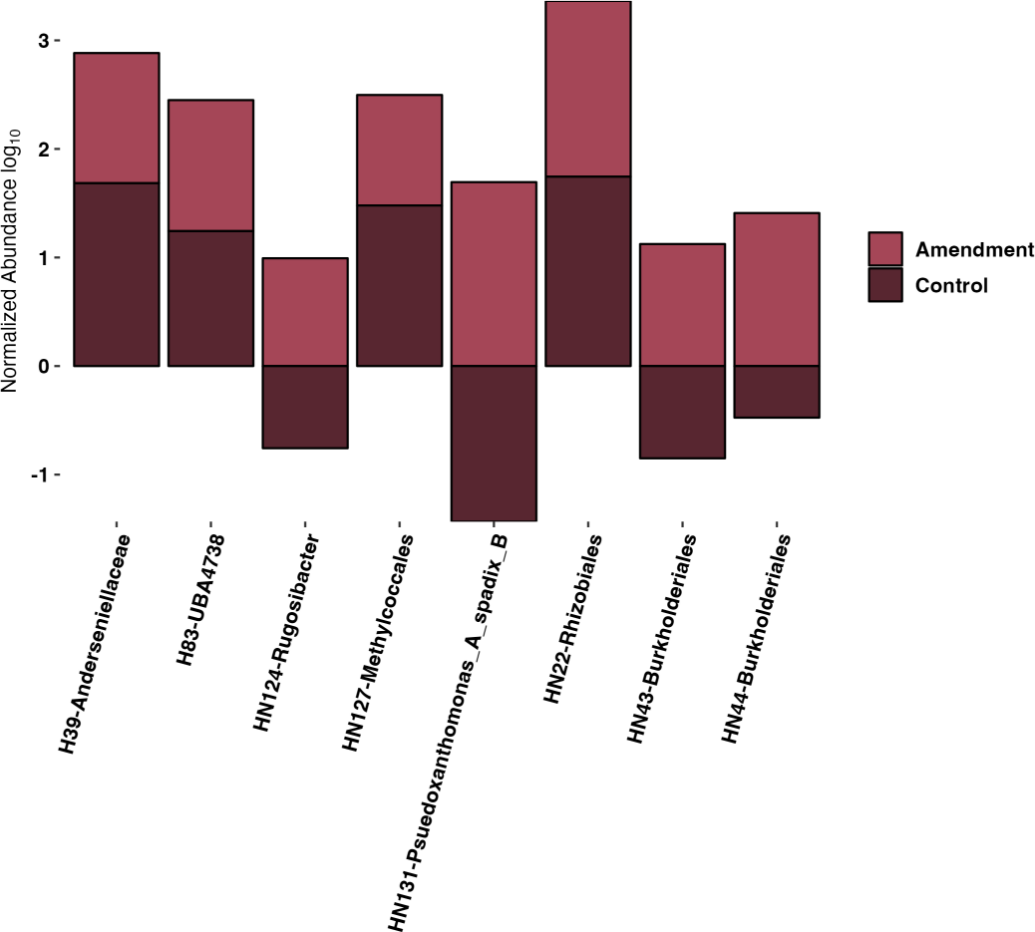
Annotation log_10_ average normalized abundance (RPKM) of selected MAGs mapped to prospective amendment and control (RPKM = number of reads/(genome length/1,000 * total number of reads/1,000,000).

Mono and di-oxygenases are responsible for activating the degradation of BTEX (14, 49, 83). Seven MAGs (HN22, HN43, HN44, HN124, HN127, HN131, H39, and H83), evident only in the light synthetic crude and light synthetic crude with nutrient amendments, contained these essential BTEX degradation genes (Fig. 2). The average normalized abundance (RPKM values) of MAGs HN43, HN44, HN124, and HN131 in the light synthetic crude with nutrient amendment were 13, 26, 10, and 49, respectively (Fig. 3). By comparison, when mapping these four MAGs (HN43, HN44, HN124, and HN131) from the light synthetic crude and nutrient amendment back to the control, an RPKM value of <1 was revealed (Fig. 3). MAGs HN127, HN22, H39, and H83 were present in a greater relative abundance in the control when compared to microcosms amended with light synthetic crude and light synthetic crude with nutrients (Fig. 3). In addition, KEGG ortholog pathways showed genes responsible for the complete degradation of intermediate products, such as catechol and benzoate, which were found dispersed in MAGs (HN7, HN22, HN43, HN44, HN70, HN77, HN124, HN131) from the light synthetic crude and nutrient amendment (Table 2).

**TABLE 2:** Genetic potential of MAGs in intermediate pathways^*a*^

	MAGs	C27	C75	C81	H2	H6	H27	H39	H46	H58	H95.2	H108	HN7	HN22	HN43	HN44	HN70	HN77	HN124	HN131
Catechol → acetyl-CoA	*dmpB*																			
	*dmpC, xylF, praB*																			
	*praC, xylH*																			
	*dmpH, xylI, nahK*																			
	*bphH, xylJ,tesE*																			
	*bphI, xylK, nahM, tesG*																			
	*bphJ, xylQ, nahO, tesF*																			
4-methylcatechol → Propanoyl-CoA	*catE*																			
	*dmpD,xylF*																			
	*mhpD*																			
	*mhpE*																			
	*mhpF*																			
Catechol → 3-oxoadipate	*catA*																			
	*catB*																			
	*catC*																			
	*pcaD, pcaL*																			
Benzoate/Methylbenzoate → Catechol/Methylcatechol	*benA-xylX*																			
	*benB-xylY*																			
	*benC-xylZ*																			
	*benD-xylL*																			
Benzoate → Pimeoyl-CoA	*aliA*																			
	*aliB*																			
	*badK*																			
	*badH*																			
	*badI*																			

^*a*^ Shaded areas (black box) reflect the presence of the gene found in each MAG.

### Aromatic degradation pathways in MAGs

**Fig 4:**
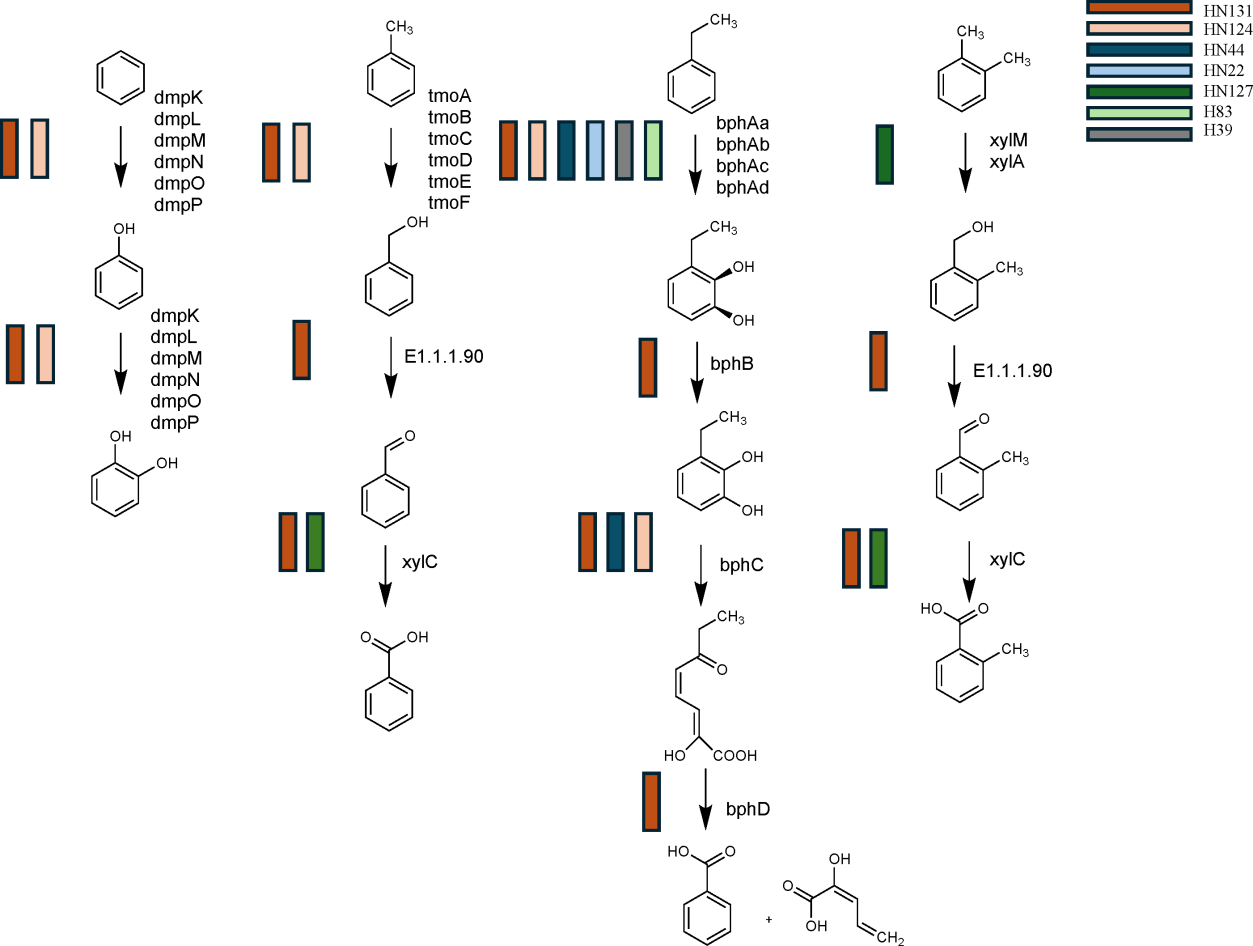
BTEX degradation pathways and affiliated MAGs constructed from KEGG ortholog pathways for BTEX.

Essential hydrocarbon degradation genes in aerobic aromatic pathways were identified only in MAGs from the light synthetic crude with nutrient amendments. Five MAGs (HN22, HN44, HN124, HN127, and HN131) found in the light synthetic crude oil with a nutrient amendment were the only MAGs that contained these key genes (*tomA1A4*, *tmoABCDEF*, *dmpKLMNOP*, *bphAbAcAd*, *xylMA*, *ndoC*, and MAHβ) responsible for the degradation of BTEX compounds (Fig. 2 and 4). Additionally, MAGs H39, H83, HN22, HN44, HN124, and HN131 contain genes (*bphA* and *bphAbAcAd*) responsible for activating the degradation of ethylbenzene. In contrast, MAGs HN44 and HN124 contain *bphC*, and HN131 possesses *bphBCD*, which completes the degradation to benzoate and 2-Oxopent-4-enoate (Fig. 2 and 4). Further, the genes that activate the xylene degradation pathway (*xylMA*) were only located in MAG HN127.

MAGs HN124 and HN131 (light synthetic crude oil with nutrient amendment) possessed all genes necessary to activate aerobic degradation pathways of all BTEX compounds and naphthalene (Fig. 2 and 4). HN131 also possessed the complete degradation pathway for benzene, toluene, ethylbenzene (via the biphenyl pathway), and 66% of the xylene pathway (Fig. 4). Further, MAGs HN124 and HN131 were the only MAGs in the experiment carrying the *dmpKLMNOP* genes, which are responsible for the complete degradation of benzene to catechol. Taxonomic analysis revealed both HN124 and HN131 were derived from the class Gammaproteobacteria with HN124 (87% complete) identified as the genus *Rugosibacter* and HN131 (97% complete) down to the species level as *Pseudoxanthomonas A spadix B*.

### Metagenome analyses of KEGG pathways document full degradation potential

Analyses of KEGG ortholog modules in metagenomes (not binned) revealed the metagenome amended with light synthetic crude oil and control contained complete degradation pathways for benzene and toluene (Table 3). Overall, both the metagenome of the control and light synthetic oil amendment only contained 66% of the xylene degradation pathway and 25% of the naphthalene degradation pathway. The light synthetic crude oil amendment metagenomic data also possessed the metabolic capabilities to degrade ethylbenzene completely. NDOs and xylene activation genes, *xylMA*, were missing from the control and light synthetic crude oil metagenomes (Table 3). Downstream intermediate pathways, such as catechol and benzoate degradation pathways, were found in all metagenomic amendments and the control, except for *benC-xylZ*, which was absent from the light synthetic crude oil amendment (Table 3).

**TABLE 3:** Metagenomic (unbinned) BTEX and intermediate pathway gene presence

	Genes	Control	Crude	Crude and nutrient
Benzene	*dmpK*			
	*dmpL*			
	*dmpM*			
	*dmpN*			
	*dmpO*			
	*dmpP*			
Toluene	*tmoA*			
	*tmoB*			
	*tmoC*			
	*tmoD*			
	*tmoE*			
	*tmoF*			
	*E1.1.1.90*			
	*xylC*			
Ethylbenzene	*bphAa*			
	*bphAb*			
	*bphAc*			
	*bphAd*			
	*bphB*			
	*bphC*			
	*bphD*			
Xylene	*xylM*			
	*xylA*			
	*E1.1.1.90*			
	*xylC*			
Naphthalene	*nahAa, nagAa, ndoR, nbzAa, dntAa*			
	*nahAb, nagAb, ndoA, nbzAb, dntAb*			
	*nahAc, ndoB, nbzAc, dntAc*			
	*nahAd, ndoC, nbzAd, dntAd*			
	*nahB, doxE*			
	*nahC*			
	*nahD*			
	*nahE*			
	*nahF*			
Catechol → acetyl-CoA	*dmpB*			
	*dmpC, xylF, praB*			
	*praC, xylH*			
	*dmpH, xylI, nahK*			
	*bphH, xylJ,tesE*			
	*bphI, xylK, nahM, tesG*			
	*bphJ, xylQ, nahO, tesF*			
4-methylcatechol → Propanoyl-CoA	*catE*			
	*dmpD,xylF*			
	*mhpD*			
	*mhpE*			
	*mhpF*			
Catechol → 3-oxoadipate	*catA*			
	*catB*			
	*catC*			
	*pcaD, pcaL*			
Benzoate/Methylbenzoate → Catechol/Methylcatechol	*benA-xylX*			
	*benB-xylY*			
	*benC-xylZ*			
	*benD-xylL*			
Benzoate → Pimeoyl-CoA	*aliA*			
	*aliB*			
	*badK*			
	*badH*			
	*badI*			

^*a*^ Shaded areas (black box) reflect the presence of the gene found in unbinned assemblies from their respective experimental microcosm.

### VOC analysis

Sediments from all microcosm treatments were analyzed to quantify the concentration of benzene, toluene, ethylbenzene, total xylene, naphthalene, and cyclohexane at the experiment’s start and completion. After 30 days, a reduction of all VOCs in the microcosms amended with light synthetic crude oil and light synthetic crude oil with nutrients was observed (Table 2). The microcosms that contained light synthetic crude oil with nutrients found benzene was reduced to below detection limits, and toluene (97.8% reduction) and ethylbenzene (66.7% reduction) were primarily reduced. All control microcosms and time points returned VOC concentrations below the detection limit, except for one replicate for toluene at day 0 (Table 4). Variability was also observed between the triplicates; therefore, a non-parametric statistical analysis was completed. Variability between the replicates could have been due to the sample heterogeneity or even the complexity of the sample extraction (the water and oil were a thin, non-mixed layer sitting atop the sediments). A Friedman test was conducted to determine if there was a significant difference in VOCs from days 0 to 30. A global Freidman analysis showed a significant difference between days 0 and 30 for all compounds other than naphthalene in both the light synthetic crude oil (*P* = 0.025, *Χ*^2^ = 12.847) and light synthetic crude oil with nutrient amendments (*P* = 0.002, *Χ*^2^ = 18.851).

**TABLE 4:** Microcosm volatile organic compound concentrations

	Amendment	Rep #	Benzene (µg/kg)	Toluene (µg/kg)	Ethylbenzene (µg/kg)	Xylene (µg/kg)	Cyclohexane (µg/kg)	Naphthalene (µg/kg)
Day 0	Biological control	1	ND^*a*^	ND	ND	ND	ND	ND
		2	ND	160	ND	ND	ND	ND
		3	ND	ND	ND	ND	ND	ND
	Crude amendment	1	4,100	21,000	9,900	40,000	7,200	2,500
		2	28,000	93,000	45,000	170,000	34,000	16,000
		3	2,400	19,000	9,900	38,000	7,800	600
	Average		11,500	44,333	21,600	82,667	16,333	6,367
	Crude and nutrient amendment	1	2,100	9,900	6,900	30,000	2,100	1,300
		2	11,000	61,000	30,000	120,000	18,000	5,700
		3	2,000	21,0000	13,000	51,000	5,900	ND
	Average		5,033	93,633	16,633	67,000	8,667	3,500
Day 30	Biological control	1	ND	ND	ND	ND	ND	ND
		2	ND	ND	ND	ND	ND	ND
		3	ND	ND	ND	ND	ND	ND
	Crude amendment	1	1,600	5,300	3,300	15,000	2,600	1,300
		2	13,000	54,000	35,000	180,000	35,000	14,000
		3	400	4,500	2,900	18,000	1,100	940
	Average		5,000	21,267	13,733	71,000	12,900	5,413
	Difference^*b*^		6,500	23,067	7,867	11,667	3,433	953
	%Reduction^*c*^		57	52	36	14	21	15
	Crude and nutrient amendment	1	ND	100	180	2,600	450	1,100
		2	ND	180	420	51,000	15,000	1,900
		3	920	5,900	16,000	90,000	16,000	16,000
	Average		ND	2,060	5,533	47,867	10,483	ND
	Difference		ND	91,573	11,100	19,133	(1,817)	ND
	%Reduction^*c*^		ND	98	67	29	(21)	ND

^*a*^ ND is not detected, as it was below the detection limit of the instrument.

^*b*^ Average of day 30 was subtracted from average of day 0.

^*c*^ Average of day 0 was divided by difference, then multiplied by 100.

## DISCUSSION

Crude oil contamination is a significant concern due to the compounds' mutagenic, toxigenic, and carcinogenic nature, with some components listed as priority pollutants by the EPA due to their harmful effects (19, 84). This study focused on BTEX and naphthalene, the more recalcitrant fractions of the light synthetic crude, and benthic sediments, a habitat where these compounds persist (46, 47, 85, 86). To date, research regarding hydrocarbon degradation in freshwater environments (8–10) has amassed much less attention than in marine environments (1–5, 17, 66, 87–89). To our knowledge, this study is one of the rare freshwater benthic sediment microcosm studies, with replicates, that examine the metagenomic potential of MAGs and connect that to actual degradation through VOC analyses. Previous sediment microcosm studies have looked at either hydrocarbon degradation potential through chemical analyses (90, 91) or single gene 16s rRNA analysis (9, 10), with two recent 16s rRNA gene studies examining both degradation potential and chemical data (92, 93).

### The addition of nutrients identifies the taxonomic diversity of experimental microcosms

Microcosms amended with light synthetic crude and nutrients contain two taxonomic groups known to degrade hydrocarbons. Xanthomonadales were enriched in microcosms amended with light synthetic oil and nutrients (Fig. 1). Xanthomonadales have been documented to be a prominent hydrocarbon-degrading taxa (13, 14). Further, the average normalized abundance of MAG HN131 (from the order Xanthomonadales, identified as *Pseudoxanthomonas A spadix B*) was high in the light synthetic crude with nutrient amendment and negligible in the control. The order Burkholderiales was also documented in both the control and light synthetic crude with nutrient amendment (Fig. 1), and this taxonomic group is also known to degrade hydrocarbons (14, 21, 83, 94). Yet, Burkholderiales MAGs within the control lacked the monooxygenases and dioxygenases responsible for BTEX degradation. In contrast, Burkholderiales MAGs documented in the light synthetic crude with nutrient amendment contained the oxygenases necessary for BTEX degradation (Fig. 4). MAGs HN43, H44, and HN124, all from the order Burkholderiales, had relatively high average normalized abundance in the light synthetic crude with nutrients amendment (absent in the control). This suggests that stimulating a wetland with light synthetic crude oil and nutrients can rapidly change the taxonomic composition, resulting in more significant numbers of rare hydrocarbon-degrading microorganisms harboring key hydrocarbon activation genes. These data coincide with previous studies that found that nutrients may be a limiting factor and increase microbial degradation potential (1, 19, 48, 87).

### Full BTEX degradation pathways identified in MAGs

MAGs that fully degraded BTEX compounds were only documented in microcosm treatments with light synthetic crude oil with nutrient amendments. Although various microorganisms have been identified as having the ability to degrade BTEX compounds, very few organisms have been noted as having the ability to degrade all BTEX compounds (32, 95, 96). One MAG, HN131, identified as *Pseudoxanthomonas A spadix B* (order Xanthomonadales), possessed the complete pathways for degrading benzene and toluene (Fig. 2 and 4). HN131 did contain the biphenyl pathway (*bphAaAbAcAd*, *bphB*, *bphC*, and *bphD*), and previous studies have noted this pathway has low substrate specificity and can degrade ethylbenzene and naphthalene (34, 92, 97). Although xylene activation genes, *xylMA*, were not located within HN131, studies have shown that xylene degradation proceeds through several pathways (32). One proposed xylene degradation pathway includes activating the pathway with *tmoABCD* (32), identified within MAG HN131 (Fig. 4), suggesting that it may possess the genetic potential to degrade all BTEX compounds. NDOs, *ndoBC* and *non ndoB*, were also documented in MAG HN131.

An outside study has found *Pseudoxanthomonas A spadix B* possesses the genetic potential to degrade benzene, toluene, and xylene (14). Other studies have noted *Pseudoxanthomonas spadix BD-a59* as having the ability to degrade all BTEX compounds (13, 98). This bacteria has even been referred to as a hydrocarbon generalist because of its ability to utilize a vast array of hydrocarbons as a sole energy source (16). Choi et al. (13) also found the presence of NDOs in *Pseudoxanthomonas spadix BD-a59* and concluded that these were used to activate the degradation of ethylbenzene. Additionally, Gutierrez et al. (16) also identified *Psuedoxanthomonas* as possessing the capabilities to degrade BTEX, phenanathrene, diesel, crude oil, *n*-tetradecane, and *n*-hexadecane. In the current study, the presence of genes *ndoBC* in MAG HN131 may also suggest the potential to break down ethylbenzene and naphthalene (Fig. 2). Therefore, evidence from this study suggests *Pseudoxanthomonas A spadix B*-HN131 is also a hydrocarbon generalist able to degrade BTEX compounds and naphthalene, identifying this microorganism as an essential member of the hydrocarbon-degrading community.

Another essential BTEX degrading MAG, classified as *Rugosibacter*-HN124 (order Burkholderiales), possessed genes that could fully degrade benzene to acetyl-CoA (Fig. 4, Table 2). Burkholderiales are noted to have the catabolic potential to degrade various hydrocarbons (14, 83, 94), which concurs with the present study’s findings. The number of monooxygenases and dioxygenases identified in *Rugosibacter*-HN124 suggests this MAG can degrade numerous aromatic compounds, including BTEX, through several pathways. Moreover, *Rugosibacter*-HN124 includes 33% of toluene and naphthalene and 25% of ethylbenzene degradation pathways. Based on the array of activation genes, complete degradation pathways, and potential to degrade intermediates, it is proposed that *Rugosibacter*-HN124 is a critical member of the consortium degrading the light synthetic crude in this study.

### Partial degradation pathways identified in MAGs

Xylene degradation genes (*xylMA* and *xylC*) were found in MAG HN127 from the experimental condition with light synthetic crude oil and nutrient amendments. This MAG was classified to the order of Methylcoccales (Fig. 4). In previous studies, these orders have been shown to play a critical role in hydrocarbon degradation (13, 94, 98, 99). Methylcoccales, a methanotroph that flourishes in environments like wetlands, as it can use methane as a sole source of carbon for energy (99). In the current study, Methylcoccales HN127 was the only MAG containing the genes necessary to activate the xylene degradation pathway, *xylMA*, suggesting it is an essential member of the consortium degrading the light synthetic crude oil. Additionally, MAG HN127 was found to have the toluene and xylene degradation gene, *xylC*, responsible for converting benzaldehyde/methylbenzaldehyde to benzoate/methylbenzoate.

MAG HN44, classified as a Burkholderiales, has the genetic potential to degrade several MAHs and PAHs. Other studies have shown Burkholderiales to include a broad range of monooxygenases and dioxygenases involved in almost all documented central ring cleavage pathways (94), similar to what was found in the current study. This MAG is another Gammaproteobacteria, as are HN 124, HN 127, and HN 131, found in the experimental conditions with nutrients. Several marine studies found Gammaproteobacteria to be a main class in hydrocarbon degradation (2, 6, 100). The array of monooxygenases and dioxygenases in these Gammaproteobacteria MAGs highlights their potential to degrade BTEX and naphthalene in the freshwater wetland studied. Nutrients are often a limiting factor in bioremediation (1, 19, 25, 87), affecting biodegradation rates by controlling the growth of hydrocarbon-degrading communities (88).

Two MAGs from the experimental conditions with only light synthetic crude oil contained partial degradation pathways. Dioxygenase, *bphA,* involved in activating the ethylbenzene degradation pathway, was found in MAGs (H39 and H83) from the experimental conditions with light synthetic crude. MAG H39 was classified as a Rhizobiales, and H83 was classified as Actinobacteria bacterium UBA4738. However, the dioxygenases (*bphAbAcAd*, *bphB*, *bphC*, and *bphD*) that complete the degradation of ethylbenzene to benzoate and 2-oxopent-4-enoate were found only in MAGs from the light synthetic crude with nutrient amendments (HN44, HN124, and HN131).

### VOC analyses connect degradation potential to function

Chemical analyses of BTEX compounds provided evidence that the genetic potential found in the MAGs may connect to functional BTEX degradation (Table 4). BTEX degradation was more prevalent in microcosms that were stimulated with additional nutrients. Consequently, this study’s experimental microcosms showed that MAGs from a freshwater wetland had relatively more hydrocarbon degradation genes when the community was exposed to both light synthetic crude oil and nutrients (Table 1). Other studies have noted that microbial degradation is driven by hydrocarbon and nitrogen (25, 87, 101) or stimulated by nutrient amendments (1, 19, 87). In the current study, the degradation of VOC compounds, combined with the increase in abundance of aerobic aromatic degradation gene potential, suggests that adding nutrients could enhance degradation.

### Experimental variability implicates high heterogeneity in wetland microcosms

High heterogeneity was observed in replicates of experimental and control microcosms. Within this study, one site was chosen to maximize the ability of the experiment to capture variability within experimental conditions. Whether looking at VOC data or even the taxonomic distribution of MAGs in the current study, vast variability was seen between replicate experimental samples. Specifically, the abundance of MAGs HN131 was negligible in one replicate but dominant in the other two replicates. As HN 131 contained numerous complete degradation pathways, vital data could have been lost if this study only had one replicate. The results of this study suggest sequencing more samples per site rather than more sites can provide a fuller understanding of the habitat.

### Treatment impacts on metagenomes

Metagenome (unbinned) analyses revealed complete hydrocarbon degradation pathways across treatments and control, supporting that nutrients may be essential when binning high-quality MAGs with hydrocarbon degradation genes. Studies monitoring legacy contaminated sites have suggested that the hydroxylated and carboxylated intermediate products can pose a greater risk to human and ecological health than the associated PAH (102, 103). Thus, the complete degradation of aromatic compounds is of extreme importance. Annotation of KEGG orthologs in metagenomes revealed complete pathways for the degradation of benzene and toluene in the control and partial pathways for xylene and naphthalene, suggesting that the MAGs associated with hydrocarbon degradation are present in the control but at an abundance too low to assemble. The resulting RPKM numbers of mapping raw reads from the control back to MAGs resulting from experimental conditions support this theory. These findings coincide with other studies (7, 9, 10, 19) that show that the wetland will respond to the addition of light synthetic crude. The data suggest that microorganisms capable of degrading hydrocarbons react rapidly to the addition of light synthetic oil and nutrients, which may be the limiting factor in hydrocarbon degradation (1, 19, 48). The current study suggests that hydrocarbon-degrading bacteria are present in this freshwater coastal wetland at an abundance too low to assemble and bin into MAGs without the addition of light synthetic crude oil and nutrients.

### Environmental implications

This microcosm study was able to determine through chemical and metagenomic analyses that the addition of nutrients can enhance hydrocarbon degradation potential. This study also documented several rate-limiting aromatic degradation genes identified in MAGs only in light synthetic crude oil and nutrients, illustrating the mechanisms the microbial communities may employ to degrade the light synthetic crude. As bioremediation strategies aim to overcome limiting factors (104), these data infer that nutrients could be a limiting factor for bioremediation in this freshwater wetland. Thus, adding nutrients could be an effective strategy in the event of a spill in this habitat. Of regional geographic interests, the current risk assessment for a possible hydrocarbon spill in the region (Mackinac Straits, USA) relies on data from marine studies and a previous river (Kalamazoo River, MI, USA) spill (45). This metagenomic study illustrated much-needed mechanistic details into how additional nutrients can increase microbial degradation potential in a nutrient-rich habitat.

## Data Availability

Raw DNA sequencing reads are publicly available at NCBI GenBank under BioProject number PRJNA761709.

## SUPPLEMENTAL MATERIAL

Tables S1 to S3 aem.01972-24-s0001.docx Data on media and DNA sequencing and assembling.

ASM does not own the copyrights to Supplemental Material that may be linked to, or accessed through, an article. The authors have granted ASM a non-exclusive, world-wide license to publish the Supplemental Material files. Please contact the corresponding author directly for reuse.
