# Supplementary material for: Nutrient amendments enrich microbial hydrocarbon degradation metagenomic potential in freshwater coastal wetland microcosm experiments
Source: Appl Environ Microbiol. 2024 Dec 9;91(1):e01972-24. doi: 10.1128/aem.01972-24 (PMC11784303; doi:10.1128/aem.01972-24)
Supplement: Tables S1 to S3 — Data on media and DNA sequencing and assembling. [file aem.01972-24-s0001.docx]

**Supplementary Material**

Supplemental Table S1. Concentration of Macronutrients in 10X Bushnell Haas Media

| Component | g/L |
| --- | --- |
| Magnesium Sulphate Heptahydrate | 4.10 |
| Calcium Chloride Dihydrate | 0.26 |
| Monopotassium Phosphate | 10.00 |
| Dipotassium Phosphate | 10.00 |
| Ammonium Nitrate | 10.00 |
| Ferric Chloride Hexahydrate | 0.83 |

Supplemental Table S2. Statistics of Megahit co-assemblies.

|  | Control | Light synthetic crude oil | Light synthetic crude oil with nutrients |  |
| --- | --- | --- | --- | --- |
| # contigs (>= 0 bp) | 6,577,659 | 6,077,704 | 6,271,901 |  |
| # contigs (>= 1000 bp) | 756,310 | 737,483 | 794,091 |  |
| # contigs (>= 5000 bp) | 21,544 | 20,496 | 25,994 |  |
| # contigs (>= 10000 bp) | 4,315 | 4,139 | 5,728 |  |
| # contigs (>= 25000 bp) | 354 | 457 | 823 |  |
| # contigs (>= 50000 bp) | 42 | 67 | 197 |  |
| Total length (>= 0 bp) | 4,292,035,488 | 4,035,388,491 | 4,271,657,132 |  |
| Total length (>= 1000 bp) | 1,379,372,032 | 1,347,452,191 | 1,513,827,954 |  |
| Total length (>= 5000 bp) | 181,370,550 | 177,539,362 | 240,134,633 |  |
| Total length (>= 10000 bp) | 67,866,360 | 70,002,178 | 106,673,364 |  |
| Total length (>= 25000 bp) | 12,884,469 | 17,994,657 | 36,902,101 |  |
| Total length (>= 50000 bp) | 2,800,185 | 5,338,538 | 16,142,051 |  |
| # contigs | 3,142,377 | 2,968,041 | 3,079,801 |  |
| Largest contig | 117,775 | 241,718 | 467,487 |  |
| Total length | 2,979,092,779 | 2,847,108,799 | 3,050,909,769 |  |
| GC (%) | 61.14 | 61.43 | 61.14 |  |
| N50 | 935 | 953 | 992 |  |
| N75 | 656 | 663 | 672 |  |
| L50 | 870,332 | 815,495 | 805767 |  |
| L75 | 1,836,902 | 1,726,373 | 1757247 |  |
| # N’s per 100 kbp | 0 | 0 | 0 |  |

Supplemental Table S3. Statistics and Taxonomy of 37 MAGs

| **Treatment** | **Domain** | **GTDB_Class** | **GTDB_Order (Lowest rank )** | **% Completion** | **% Contamination** | **GC Content** | **Total Length (bp)** | **N 50** | **# of Contigs** | **MAG** | **RPKM** |
| --- | --- | --- | --- | --- | --- | --- | --- | --- | --- | --- | --- |
| Crude & Nutrient | Bacteria | Alphaproteobacteria | Rhizobiales (g_Rhodopseudomonas) | 97.65 | 1.88 | 64.63 | 3736939 | 19204 | 280 | HN_56 | 18.17 |
| Crude & Nutrient | Bacteria | Alphaproteobacteria | Rhizobiales (g_QKVK01) | 78.13 | 1.72 | 64.33 | 2958426 | 6595 | 510 | HN_22 | 39.24 |
| Crude & Nutrient | Bacteria | Chlorobia | Chlorobiales (f_Chlorobiaceae) | 96.55 | 0 | 55.86 | 2050681 | 11497 | 237 | HN_88 | 21.89 |
| Crude & Nutrient | Bacteria | Cyanobacteriia | Synechococcales (s_WH-5701 sp002252635) | 91.22 | 0 | 67.03 | 2325353 | 7868 | 349 | HN_115 | 19.25 |
| Crude & Nutrient | Bacteria | Desulfobacteria | Desulfobacterales (f_Desulfosarcinaceae_B) | 81.03 | 1.72 | 62.73 | 3788656 | 6979 | 618 | HN_77 | 30.51 |
| Crude & Nutrient | Bacteria | Gammaproteobacteria | Xanthomonadales (s_Pseudoxanthomonas_A spadix_B) | 96.55 | 0 | 67.72 | 3501695 | 50010 | 116 | HN_131 | 43.30 |
| Crude & Nutrient | Bacteria | Gammaproteobacteria | Methylococcales (g_KS41) | 96.55 | 0 | 47.09 | 3784969 | 11488 | 423 | HN_127 | 10.96 |
| Crude & Nutrient | Bacteria | Gammaproteobacteria | Burkholderiales (f_Burkholderiaceae) | 90.05 | 0.16 | 65.25 | 4191812 | 19551 | 347 | HN_7 | 30.61 |
| Crude & Nutrient | Bacteria | Gammaproteobacteria | Burkholderiales (g_Rugosibacter) | 86.77 | 3.45 | 54.03 | 2141735 | 11192 | 256 | HN_124 | 8.86 |
| Crude & Nutrient | Bacteria | Gammaproteobacteria | Burkholderiales (s_Janthinobacterium svalbardensis) | 82.76 | 3.45 | 62.97 | 5066438 | 7232 | 799 | HN_43 | 17.29 |
| Crude & Nutrient | Bacteria | Gammaproteobacteria | Burkholderiales (g_Acidovorax_B) | 76.11 | 1.88 | 65.08 | 3741622 | 11193 | 445 | HN_44 | 26.65 |
| Crude & Nutrient | Archaea | Methanobacteria | Methanobacteriales(f_Methanobacteriaceae) | 96.19 | 1.87 | 38.37 | 1813028 | 23973 | 108 | HN_159 | 11.10 |
| Crude & Nutrient | Archaea | Methanocellia | Methanocellales (f_Methanocellaceae) | 82.17 | 1.87 | 58.96 | 2321101 | 7081 | 371 | HN_70 | 13.65 |
| Crude | Bacteria | Alphaproteobacteria | Rhodospirillales (g_Phaeospirillum) | 84.8 | 3.45 | 64.38 | 3615292 | 14010 | 365 | H_41 | 13.23 |
| Crude | Bacteria | Alphaproteobacteria | Rhizobiales (f_Anderseniellaceae) | 82.84 | 0 | 64.34 | 3108754 | 6912 | 524 | H_39 | 18.29 |
| Crude | Bacteria | Alphaproteobacteria | Rhizobiales (g_Methyloceanibacter) | 77.51 | 3.45 | 64.31 | 2116898 | 14508 | 210 | H_34 | 24.14 |
| Crude | Bacteria | Anaerolineae | Anaerolineales (f_Anaerolineaceae) | 94.04 | 1.02 | 60.35 | 4889539 | 11243 | 581 | H_95_2 | 6.88 |
| Crude | Bacteria | Chlorobia | Chlorobiales (g_Chlorobium) | 94.83 | 1.88 | 55.65 | 1959940 | 8963 | 265 | H_58 | 47.26 |
| Crude | Bacteria | Chlorobia | Chlorobiales (g_Chlorobaculum) | 84.48 | 0 | 57.87 | 1969525 | 7055 | 320 | H_2 | 75.83 |
| Crude | Bacteria | Gammaproteobacteria | Diplorickettsiales (g_Rickettsiella) | 100 | 0 | 40.39 | 1502998 | 50570 | 56 | H_46 | 8.46 |
| Crude | Bacteria | Gammaproteobacteria | Chromatiales (f_Chromatiaceae) | 95.69 | 1.55 | 67.45 | 3371126 | 6389 | 594 | H_108 | 11.09 |
| Crude | Bacteria | Gammaproteobacteria | Burkholderiales (f_Burkholderiaceae) | 94.33 | 1.33 | 65.67 | 3969606 | 23382 | 248 | H_6 | 17.84 |
| Crude | Bacteria | Gammaproteobacteria | Methylococcales (g_KS41) | 91.95 | 0 | 46.85 | 3354143 | 9313 | 441 | H_64 | 8.78 |
| Crude | Bacteria | Gammaproteobacteria | Burkholderiales (g_Dechloromonas) | 82.07 | 0 | 62.73 | 2625824 | 7942 | 385 | H_56 | 19.48 |
| Crude | Bacteria | Gracilibacteria | BD1-5 (f_UBA6164) | 91.22 | 0.86 | 26.28 | 1265894 | 127475 | 24 | H_60 | 239.63 |
| Crude | Archaea | Methanocellia | Methanocellias (f_Methanocellaceae) | 77.57 | 0.93 | 58.74 | 2435250 | 6870 | 404 | H_27 | 16.28 |
| Crude | Bacteria | UBA4738 | UBA4738 | 76.49 | 1.49 | 69.46 | 2355947 | 24791 | 146 | H_83 | 10.25 |
| Control | Bacteria | Alphaproteobacteria | Rhizobiales (g_Methyloceanibacter) | 79.86 | 0.16 | 64.10 | 2274282 | 171111 | 212 | C_42 | 71.47 |
| Control | Bacteria | Bacteroidia | Bacteroidales (g_LD21) | 76.96 | 0 | 39.90 | 4112383 | 6476 | 724 | C_4 | 16.75 |
| Control | Bacteria | Gammaproteobacteria | Burkholderiales (f_Burkholderiaceae) | 94.2 | 1.72 | 65.35 | 4329384 | 27859 | 304 | C_81 | 75.00 |
| Control | Bacteria | Gammaproteobacteria | Methyloccales (g_KS41) | 89.66 | 3.45 | 47.13 | 3469506 | 8997 | 472 | C_11 | 26.19 |
| Control | Bacteria | Gammaproteobacteria | Chromatiales (f_Chromatiaceae) | 86.21 | 0.16 | 67.61 | 3068318 | 7457 | 485 | C_75 | 39.23 |
| Control | Bacteria | Gammaproteobacteria | Burkholderiales (g_Dechloromonas) | 76.78 | 3.06 | 62.58 | 3161771 | 7918 | 496 | C_98 | 34.58 |
| Control | Bacteria | Gammaproteobacteria | Diplorickettsiales (s_Rickettsiella isopodorum) | 75.81 | 3.45 | 37.18 | 1231525 | 6670 | 208 | C_62 | 16.58 |
| Control | Bacteria | Negativicutes | UBA1444 (g_UBA1444) | 77.82 | 2.59 | 50.62 | 1869772 | 5020 | 383 | C_26 | 18.08 |
